# PANoptosis-mediated mechanisms underlying AST elevation in *Talaromyces marneffei* infection

**DOI:** 10.1371/journal.pntd.0013443

**Published:** 2025-09-02

**Authors:** Wudi Wei, Baili Zhan, Lixiang Chen, Gang Wang, Xiuli Bao, Xiaotao He, Meng Zhang, Xiaoting Xie, Weihong Huang, Zhiman Xie, Junjun Jiang, Hao Liang, Li Ye

**Affiliations:** 1 Joint Laboratory for Emerging Infectious Diseases in China (Guangxi)-ASEAN, Life Sciences Institute, Guangxi Medical University, Guangxi. China; 2 Guangxi Key Laboratory of AlDS Prevention and Treatment, School of Public Health, Guangxi Medical University, Guangxi, China; 3 The Fourth People’s Hospital of Nanning, Guangxi, China; University of San Martin de Porres Faculty of Medicine: Universidad de San Martin de Porres Facultad de Medicina Humana, PERU

## Abstract

**Background:**

*Talaromyces marneffei* (*T. marneffei*), a life-threatening opportunistic fungal pathogen, is endemic to Southeast Asia. Although elevated aspartate aminotransferase (AST) levels are commonly observed in infected individuals, the origin and mechanism of this phenomenon remain unclear. This study aimed to determine whether AST is a specific clinical indicator of *T. marneffei* infection and to investigate the underlying mechanisms associated with tissue damage and cell death.

**Methods:**

We retrospectively analyzed clinical and laboratory data of HIV/AIDS patients with or without *T. marneffei* infection from the Fourth People’s Hospital of Nanning, Guangxi. A murine model of *T. marneffei* infection was constructed to investigate AST distribution in tissues. Additionally, PANoptosis-related proteins expression and inflammatory cytokines levels were assessed using ELISA, qPCR, and Western blotting.

**Results:**

Patients with HIV/ *T. marneffei* co-infection demonstrated significantly higher serum AST levels than HIV-only individuals, which declined following antifungal therapy. In infected mice, AST levels increased progressively in plasma and organs, with hepatic levels elevated throughout days 7, 14, and 21 post-infection. The liver exhibited the highest AST concentration, while the spleen showed the greatest fold increase. PANoptosis markers, including P-RIP, RIPK1, RIPK3, P-MLKL, GSDME, GSDMD, cleaved GSDMD, caspase-3, caspase-7, caspase-8, caspase-9, were markedly upregulated in liver tissues. Concurrently, proinflammatory cytokines Tnf-α, Il-1β, and Il-18 were consistently elevated in the liver but suppressed in the spleen, indicating organ-specific immune responses.

**Conclusions:**

Our findings demonstrate that *T. marneffei* infection triggers PANoptosis- mediated hepatocyte death and hepatic inflammatory activation, which contributes to AST elevation. AST may serve as a potential auxiliary biomarker for early diagnosis and therapeutic monitoring in Talaromycosis.

## Author summary

*Talaromyces marneffei* (*T. marneffei*) is a life-threatening fungal pathogen. Elevated serum aspartate aminotransferase (AST) is commonly observed in patients with *T. marneffei* infections, though its pathogenic mechanisms remain undefined. This study aimed to determine whether AST could serve as a specific indicator of *T. marneffei* infection and to elucidate its molecular mechanisms of elevation.

In this study, through retrospective analysis of clinical data from HIV-positive patients with and without *T. marneffei* co-infection. Our findings demonstrated significantly elevated AST levels in co-infected individuals, with subsequent normalization following antifungal therapy. To further elucidate the mechanisms underlying AST elevation, we constructed a murine model of *T. marneffei* infection. The research found that a time-dependent increase in hepatic AST levels concomitant with upregulated expression of PANoptosis factors and inflammatory cytokines in liver tissue. These findings demonstrate that *T. marneffei* infection induces hepatocyte death and hepatic inflammation through activation of the PANoptosis pathway, resulting in subsequent AST release into circulation. AST may serve as a potential auxiliary biomarker for early diagnosis and therapeutic monitoring of *T. marneffei* infection, demonstrating considerable clinical utility.

## Introduction

*Talaromyces marneffei (T. marneffei)*, a thermally dimorphic fungus, is an opportunistic pathogen responsible for life-threatening systemic infections. Recognized as a priority fungal pathogen by the World Health Organization [1]. Notably, Talaromycosis ranks among the leading causes of death in individuals with Human Immunodeficiency Virus/Acquired Immunodeficiency Syndrome (HIV/AIDS), accounting for 17.5% of AIDS-related mortality and representing the third most common opportunistic infection in this population [2]. By the end of 2023, an estimated 39.9 million people were living with HIV globally [3]. Globally, the HIV/AIDS pandemic has led to a concomitant increase in *T. marneffei* infection. As of mid-2022, over 288,000 cases of Talaromycosis had been reported across 34 countries and regions [2]. Talaromycosis is characterized by a prolonged diagnostic period and a high mortality rate [4,5]. Although fungal culture remains the gold standard for the clinical diagnosis of Talaromycosis, it is time-consuming, typically requiring around ten days. Alarmingly, Talaromycosis carries a near-100% mortality rate in untreated individuals [6], with case fatality rates persisting at 24.25% despite current antifungal treatments [7]. Thus, establishing accurate and rapid diagnosis, along with effective treatment, is essential for reducing mortality and improving survival outcomes in individuals with HIV/AIDS.

Hepatosplenomegaly is a common clinical manifestation in patients with Talaromycosis [8–10]. Elevated levels of aspartate aminotransferase (AST) have been frequently observed in individuals infected with *T. marneffei*, suggesting potential hepatic involvement [11,12]. AST is predominantly localized in the liver and myocardium, and its release into the bloodstream generally reflects hepatocellular injury. AST, as a widely used biomarker of liver damage, shows a marked decrease following antifungal therapy in patients with Talaromycosis [11]. Therefore, dynamic monitoring of AST levels may serve as both a diagnosis adjunct for *T. marneffei* infection and a therapeutic response indicator. However, the molecular mechanisms underlying AST elevation in *T. marneffei* infection remain poorly understood.

While elevated AST levels are typically indicative of substantial cell death, the underlying modes of cell death are diverse and mechanistically distinct. Cell death is a highly regulated and heterogeneous biological phenomenon, encompassing several distinct pathways including apoptosis, necroptosis, pyroptosis, ferroptosis and so on. Previous studies have shown that *T. marneffei* induces pyroptosis in macrophages via the caspase-1 pathway [13]. In addition, macrophages also play a pivotal role in anti-fungal immunity by orchestrating pro-inflammatory responses against *T. marneffei*, through secretion of key cytokines including tumor necrosis factor-alpha (TNF-α) and interleukin-12 (IL-12) [14,15]. However, excessive cytokine production may lead to cell death and tissue injury [16]. PANoptosis, a recently described form of programmed cell death, integrates features of apoptosis, necroptosis, and pyroptosis, and has been implicated in various pathological conditions [17–19]. At present, the relationship between *T. marneffei*-induced liver injury and PANoptosis unestablished. Therefore, investigating this relationship may provide insights into novel therapeutic strategies for managing Talaromycosis.

In this study, we conducted a retrospective analysis of epidemiological data to evaluate the association between *T. marneffei* infection and serum AST levels. Second, a Balb/c mouse model of *T. marneffei* infection was conducted to investigate the underlying mechanisms of AST elevation. Finally, the potential involvement of PANoptosis in *T. marneffei*-induced hepatic injury was examined, with the aim of assessing the utility of AST as a potential biomarker for early diagnosis and therapeutic evaluation, and providing a theoretical basis for the development of novel diagnostic and therapeutic strategies for Talaromycosis.

## Materials and methods

### Ethics statement

This study was approved by the Human Research Ethics Committee of Guangxi Medical University (Ethical Review No. KY0006). This study utilized anonymized data for analysis, and verbal informed consent was obtained from all participating patients. The animal experimental protocol of this study was approved by the Animal Ethics Committee of Guangxi Medical University (Ethical Review No. 202006001).

### Population based data

#### Data sources.

Demographic characteristics, clinical laboratory parameters, and treatment outcomes of hospitalized patients with HIV infection, HIV/*T. marneffei* co-infection, and HIV/other opportunistic infections at Nanning Fourth People’s Hospital from 2013 to 2023 were systematically collected using EpiData 3.1. The inclusion criteria were: (1) age ≥ 18 years and (2) gold-standard-confirmed HIV infection, either alone, with *T. marneffei*, or with other opportunistic infections. Exclusion criteria were: (1) more than 50% of the clinical data is missing and (2) duplicated case records.

#### Statistical methods.

In this study, the mean ± standard deviation (x̄ ± SD) is used to describe data that conform to a normal distribution, while the median and interquartile range are used to describe data that do not conform to a normal distribution. Student’s t-tests were used for analysis of metric data that conform to a normal distribution and have equal variances, while non-parametric tests are applied for data that do not follow a normal distribution. A *P*-value of less than 0.05 denotes statistically significant differences. All statistical analyses are performed using SPSS version 21.0.

### Animal experiments

#### Reagents.

100 x Penicillin-Streptomycin mixed solution (P1400), YPD (LA0220), PDA (P8931), 4% tissue cell fixative (P1110) were purchased from Beijing Solarbio Science & Technology Co., Ltd., Sodium pentobarbital (CAS: 4390-16-3) was obtained from Sigma-Aldrich (Merck), Caspase-3 antibody (ab184787), Caspase-7 antibody (ab256469), Caspase-8 antibody (ab108333), Caspase-9 antibody (ab202068), Gsdme antibody (ab215191), Caspase-1 antibody (ab207802), Caspase-11 antibody (ab180673), Gsdmd antibody (ab209845) were purchased from Abcam (UK), Cleaved-Gsdmd antibody (cst), phospho-MLKL antibody (cst37333), phospho-RIP antibody (cst31122), anti-β-actin antibody (8H10D10) were purchased from Cell Signaling Technology (USA), 680 RD Donkey anti-mouse secondary antibody (926-68072), 800 CW Donkey anti-rabbit secondary antibody (926-32213) were purchased from LI-COR (USA). All primary antibodies were diluted at a ratio of 1:1000, and all secondary antibodies were diluted at a ratio of 1:15000.

#### Strains and experimental animals.

*T. marneffei* (ATCC18224) was obtained from American Type Culture Collection (ATCC). Female Balb/c mice, SPF grade, were purchased from Sibeifu Biotechnology Co., Ltd. (Beijing, China) (License No.: SCXK [Jing] 2019-0010).

#### Fungal culture.

*T. marneffei* was cultured on potato dextrose agar medium, which was autoclaved and supplemented with 1% Penicillin-streptomycin mixed solution. The medium was incubated in a 25°C incubator for 2–3 weeks. The conidia from the surface were collected using sterile PBS and filtered through sterile glass wool to obtain the fungal suspension. The 100 μL of the fungal suspension were added to DMEM culture medium and incubated at 37°C for 24–48 hours to assess for contamination. If no contamination was observed, the fungal suspension was mixed evenly, counted, aliquoted, and stored at 4°C for future use.

#### Animal model construction.

Forty mice were randomly divided into two groups: (1) an infection group receiving 200 μL of *T. marneffei* spore suspension (10⁸ CFU/mL) via intravenous tail injection, and (2) a healthy control group receiving an equal volume of physiological saline. At days 7, 14, and 21 post-injection, the mice were anesthetized via intraperitoneal injection of pentobarbital sodium solution, followed by the collection of blood and organs (heart, liver, spleen, lungs and kidneys).

#### Enzyme-Linked Immunosorbent Assay (ELISA).

Blood samples were collected from mice using the enucleation method followed by centrifugation to obtain plasma. Cardiac, hepatic, splenic, pulmonary, and renal tissues were excised from mice and immediately perfused with normal saline to remove residual blood. Tissue specimens were then homogenized in lysis buffer using a mechanical homogenizer, followed by centrifugation at 4°C to obtain clarified tissue lysates. The AST levels in plasma and tissue lysates were determined using a commercially available quantitative sandwich ELISA kit, with all procedures performed in strict accordance with the manufacturer’s protocol.

#### Reverse transcription quantitative PCR (RT-qPCR).

In this study, primers were designed using the PrimerBank (https://pga.mgh.harvard.edu/primerbank/) and the National Coalition Building Institute (NCBI: https://www.ncbi.nlm.nih.gov/gene/) databases, with the primer sequences provided in S1 Table. Total RNA was extracted from tissue samples using the Trizol reagent, followed by reverse transcription and real-time quantitative reverse transcription polymerase chain reaction (RT-qPCR) using the Takara Reverse Transcription Kit (Takara, RR036A) and the GeneStar PCR Master Mix (GeneSta, A303).

#### Western blotting.

Total proteins were extracted from mouse organ tissues after sample weighing. Protein concentrations were quantified using the BCA Protein Assay Kit (Beyotime). The tissue proteins were separated by SDS-PAGE (BOSTER) and transferred onto a PVDF membrane (Bio-Rad). The PVDF membrane was blocked with 5% non-fat milk at room temperature for one hour, followed by three TBST washes. The membrane was incubated with the primary antibody (diluted at 1:1000) overnight at 4°C. After removing nonspecifically bound antibodies through TBST washes, membrane was incubated with the corresponding secondary antibody (1:15000 dilution) for 1 h at room temperature, followed by additional washes to remove any unbound secondary antibody. Protein signals on the captured membrane were detected using the Odyssey CLX dual-color infrared laser imaging system (LI-COR Biosciences).

#### Statistical methods.

Results were visualized using GraphPad Prism 8. The target genes expression levels were calculated using the ΔΔCT method. ELISA and PCR results were expressed as x ± S, and differences among groups were assessed using ANOVA. Post-hoc comparisons were conducted using the Least Significant Difference (LSD) test for pairwise comparisons. The significance level (α) was set at 0.05, and a *P*-value less than 0.05 was considered to indicate statistically significant differences.

## Results

### Baseline characteristics of the study population

A total of 125 samples were included in this study, with the majority of participants (80.00%) aged between 20 and 60 years. Patients with *T. marneffei* infection accounted for 52.80% (66 cases). Among all participants, there were 87 males and 38 females. The ethnic composition was predominantly Han (57 cases) and Zhuang (61 cases). The 79 patients were engaged in occupations as farmers and labor, representing a proportion of 63.20%.

The cohort demonstrated severe immunosuppression, with 90 cases (72.00%) showing CD4 cell counts (CD4) less than 100 cells/μL, and 102 cases (81.60%) with CD4 less than 200 cells/μL (S2 Table). Furthermore, among the 102 cases with CD4 < 200 cells/μL, 62 (60.78%) had HIV/*T. marneffei* co-infection. Among the 90 cases with CD4 < 100 cells/μL, 59 (65.56%) were co-infected with HIV and *T. marneffei* (S2 Table).

### Comparative analysis of laboratory indicators in HIV and HIV/ *T. marneffei* Groups

Patients were divided into two groups: HIV and HIV/ *T. marneffei*, to compare various laboratory parameters. The HIV/ *T. marneffei* group demonstrated significantly depressed hematologic parameters including CD4, red blood cell count (RBC), white blood cell count (WBC), monocyte count (Mono), lymphocyte count (Lym), platelet count (PLT), and hemoglobin (Hb) (Table 1, *P* < 0.05). Hepatic function analysis revealed significantly reduced total protein (TP) and albumin (ALB) levels, with concurrent elevations in AST, alanine aminotransferase (ALT), and the AST/ALT ratio, as shown in Table 1 (*P* < 0.05).

**Table 1:** Levels of laboratory test indicators in patients infected with HIV and HIV/*T. marneffei* group before treated.

	HIV (n = 59)	HIV/*T. marneffei* (n = 66)	U/T	*P*
AST (U/L)	27.30 (18.50, 35.00)	60 (37.00, 110.10)	3215.500	< 0.0001
ALT (U/L)	19.10 (10.70, 33.00)	32.70 (19.10, 60.90)	2793.500	< 0.0001
AST/ALT	1.43 (1.20, 1.86)	1.67 (1.45, 2.38)	2604.500	< 0.0001
CD3 (cells/μL)	619.00 (424.00, 897.00)	284 (188.00, 537.00)	827.000	< 0.0001
CD4 (cells/µL)	71.00 (20.00, 241.00)	19.00 (10.00, 40.00)	938.500	< 0.0001
CD8 (cells/µL)	459.00 (317.00, 797.00)	273.00 (147.00, 443.00)	947.500	< 0.0001
CD4/CD8	0.16 (0.05, 0.75)	0.08 (0.04, 0.18)	1432.500	0.016
TP (g/L)	72.00 (62.20, 82.00)	66.60 (57.90, 71.80)	1215.500	0.001
ALB (g/L)	33.60 (31.00, 38.20)	29.00 (24.90, 33.30)	950.500	< 0.0001
TBIL (μmol/L)	7.89 (5.15, 10.18)	8.55 (5.83, 12.50)	2368.500	0.008
DBIL (μmol/L)	3.17 (2.31, 4.05)	4.1 (2.37, 6.81)	2522.500	0.001
TBA (μmol/L)	6.10 (4.45, 14.10)	10.00 (5.50, 22.25)	2334.000	0.004
LDH (U/L)	245.00 (201.50, 323.00)	335.00 (230.45, 494.65)	1129.500	0.003
WBC (× 10^12^/L)	5.10 (4.00, 6.00)	2.50 (2.10, 3.50)	1307.500	0.002
RBC (× 10^12^/L)	3.58 ± 0.84	3.26 ± 0.90	1.999	0.048
Hb (g/L)	98.29 ± 23.44	85.16 ± 21.44	3.245	0.002
PLT (× 10⁹/L)	217.00 (139.00, 285.00)	146.00 (96.00, 207.00)	1258.500	0.001
Mono (× 10^9^/L)	0.40 (0.20, 0.60)	0.20 (0.10, 0.30)	804.000	< 0.0001
Lym (× 10^9^/L)	1.29 ± 0.84	0.55 ± 0.41	6.246	< 0.0001

*Abbreviations: CD3, CD3 lymphocyte count; CD8, CD8 T-cells count; CD4/CD8, CD4-to-CD8 ratio; TBIL, total bilirubin; DBIL, direct bilirubin; TBA, total bile acids; LDH, lactate dehydrogenase. Mean ± SD, median (Q1, Q3).

To specifically evaluate the association between *T. marneffei* infection and AST variation, propensity score matching (PSM) was performed to adjust for potential confounding variables. including CD4 count and gender. Post-matching analysis revealed no significant intergroup differences in in CD4, RBC, WBC, Lym, and Hb (*P* > 0.05). However, ALB levels remained significantly lower in the HIV/ *T. marneffei* group (26.70, IQR:29.35, 34.10), while AST (47.50, IQR:30.55, 90.20) and ALT (28.00, IQR:19.03, 62.90) levels remained significantly elevated, as shown in Table 2 (*P* < 0.05).

**Table 2:** Levels of laboratory test indicators in patients infected with HIV and HIV/*T. marneffei* group before treatment (after PSM).

	HIV(n = 29)	HIV/*T. marneffei* (n = 29)	U/T	*P*
AST (U/L)	27.30 (22.55, 34.90)	47.50 (30.55, 90.20)	1100.000	< 0.0001
ALT (U/L)	20.00 (12.35,32.50)	28.00 (19.03, 62.90)	940.500	0.001
AST/ALT	1.43 (1.20, 1.86)	1.67 (1.45, 2.38)	806.500	0.074
CD3 (cells/μL)	569.00 (324.50, 846.50)	421.00 (227.00, 676.00)	521.000	0.077
CD4 (cells/µL)	50.00 (14.00, 73.00)	35.00 (15.75, 54.25)	674.000	0.910
CD8 (cells/µL)	459.00 (292.50, 743.00)	364.50 (175.50, 540.00)	539	0.116
CD4/CD8	0.12 (0.05, 0.19)	0.095 (0.06, 0.19)	786.000	0.272
TP (g/L)	71.60 (62.50, 82.30)	68.45 (57.38, 73.18)	511.5	0.124
ALB (g/L)	32.30 (30.95, 36.05)	26.70 (29.35, 34.10)	440.000	0.019
TBIL (μmol/L)	7.89 (5.15, 10.18)	8.55 (5.83, 12.50)	774.500	0.154
DBIL (μmol/L)	3.17 (2.31, 4.05)	4.10 (2.37, 6.81)	827.500	0.043
TBA (μmol/L)	6.03 (4.45, 14.10)	9.55 (4.58, 28.35)	847.500	0.012
LDH (U/L)	245.00 (187.55, 314.10)	304.00 (197.08, 489.63)	382.000	0.075
WBC (× 10^12^/L)	4.80 (3.70, 5.90)	3.65 (2.50, 5.98)	0.200	0.656
RBC (× 10^12^/L)	3.63 ± 0.92	3.44 ± 0.97	0.881	0.381
Hb (g/L)	96.86 ± 22.25	90.00 ± 20.40	1.373	0.174
PLT (× 10⁹/L)	217.00 (139.00, 285.00)	146.00 (96.00, 207.00)	466.000	0.027
Mono (× 10^9^/L)	0.40 (0.20, 0.60)	0.20 (0.10, 0.40)	404.500	0.004
Lym (× 10^9^/L)	0.96 ± 0.60	0.73 ± 0.46	1.787	0.079

*Mean ± SD, Median (Q1, Q3).

### AST levels in patients with HIV/ *T. marneffei* Co-infection

**Fig 1:**
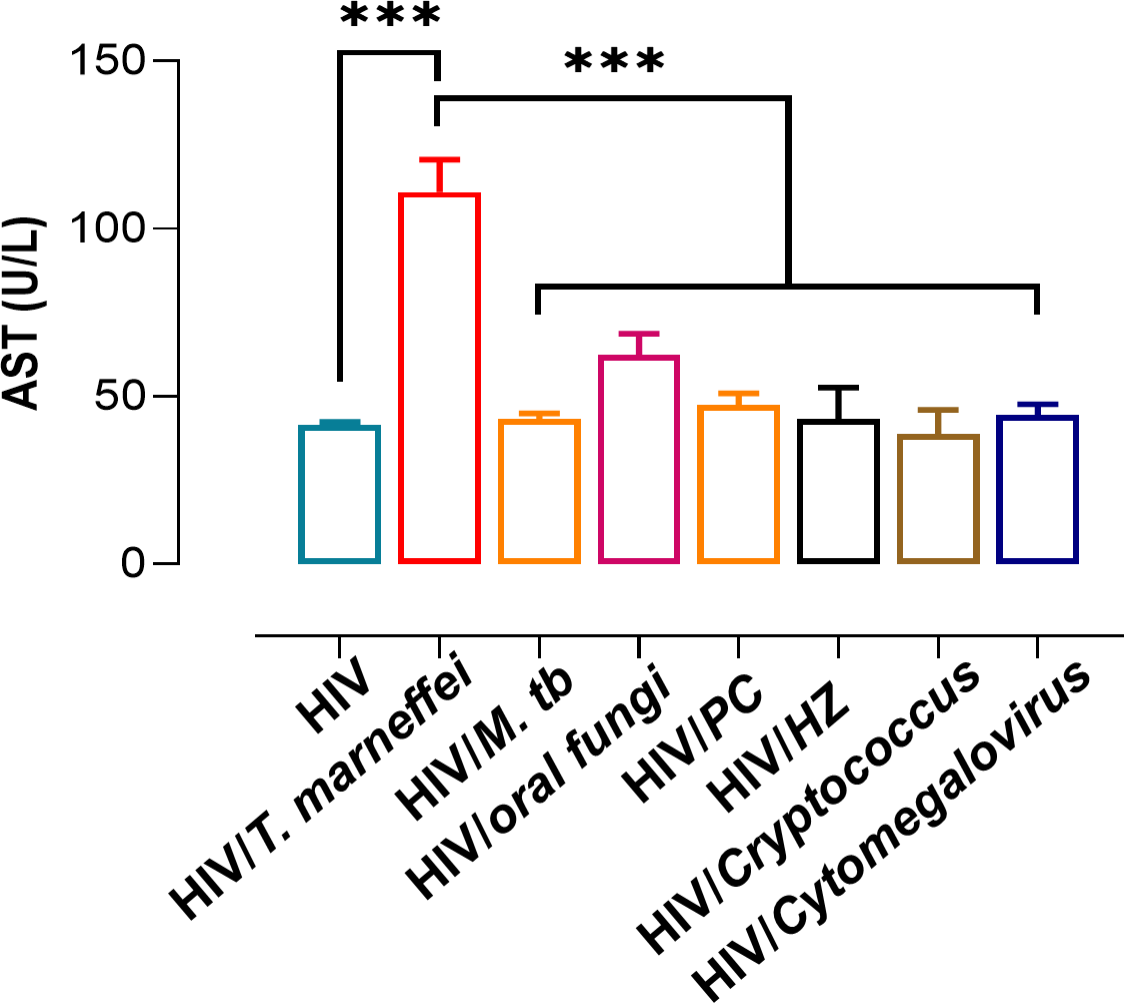
AST levels in pre-treat HIV and HIV co-infected patients with other diseases. *, **P* *< 0.05; **, *P* < 0.01; ***, *P* < 0.001; ****, **P* *< 0.0001; ns, no statistical significance. Abbreviation: *M.tb*, *Mycobacterium tuberculosis*; PC, *Pneumocystis carinii*; HZ, Herpes Zoster. Error bars: SEM. N = 11705.

Clinical data from patients with HIV monoinfection, HIV/*T. marneffei* co-infection, and HIV co-infected with other pathogens were collected and analyzed. The HIV/ *T. marneffei* group exhibited significantly higher plasma AST levels compared to other groups (Fig 1).

**Fig 2:**
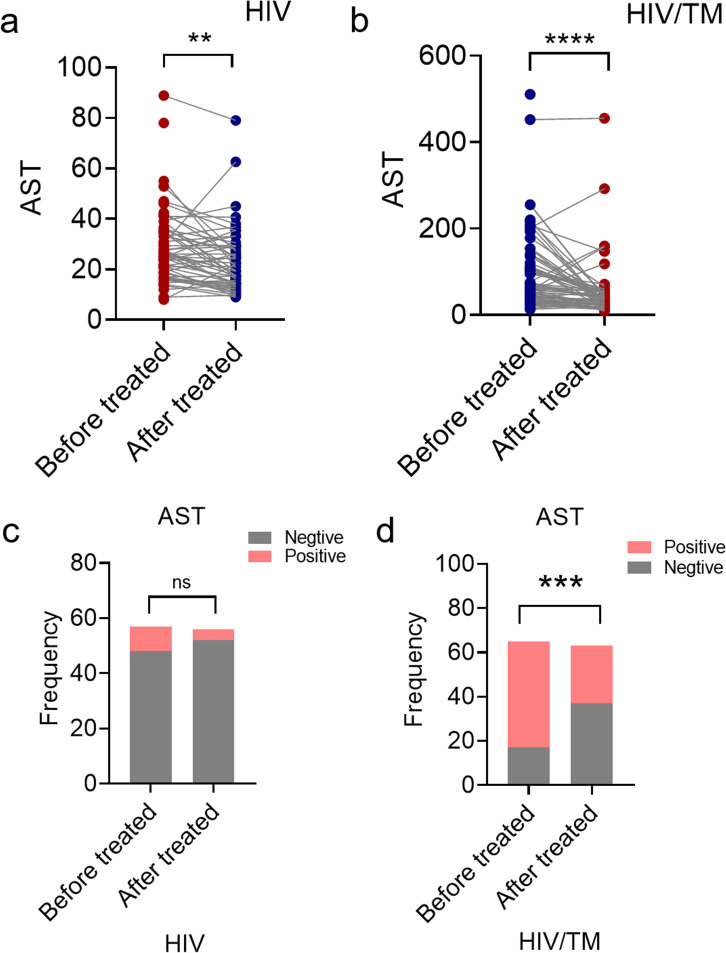
Comparison of AST levels in HIV and HIV/*T. marneffei* infected patients before and after treated. **(a)** The levels of AST expression before and after treatment in patients with HIV infection. **(b)** The levels of AST expression before and after treatment in patients with HIV/*T. marneffei* co-infection. **(c)** The levels of AST expression before and after treatment in patients with HIV infection. **(d)** The levels of AST expression before and after treatment in patients with HIV/*T. marneffei* co-infection. **(c - d)** The y-axis indicates the frequency of AST levels above or below the normal range before and after treatment. *, **P* *< 0.05; **, *P* < 0.01; ***, *P* < 0.001; ****, *P* < 0.0001; ns, no statistical significance.

To investigate the potential association between *T. marneffei* infection and elevated AST levels, we examined the changes in AST levels following antifungal therapy. A comparative analysis of AST levels was performed between hospital admission and pre-discharge timepoints in two patient groups: the HIV group and the HIV/*T. marneffei* coinfection group. Between the groups, HIV patients received treatment for 2–46 days (mean: 12.36 days), while HIV/ *T. marneffei* co-infected patients were treated for 2–71 days (mean: 15.95 days). Paired t-test analysis revealed that AST levels decreased significantly after treatment in both the HIV and HIV/ *T. marneffei* groups (Fig 2a and 2b).

Subsequently, AST levels data were categorized as either normal or abnormal according to clinical reference standards, with statistical comparisons performed using chi-square tests (Fig 2c–2d). In the HIV group, pretreatment evaluation revealed normal AST levels in 48 cases, with abnormal levels observed in 9 patients. After treatment, the number of patients with normal AST increased to 52, while those with abnormal levels decreased to 4. In the HIV/ *T. marneffei* group, pretreatment evaluation revealed normal AST levels in 17 cases, with abnormal levels observed in 48 cases. Following treatment, the number of patients with normal AST increased to 37, and the number with abnormal AST decreased to 26.

### Elevated AST levels in plasma and tissues of mice infected with *T. marneffei*

**Fig 3:**
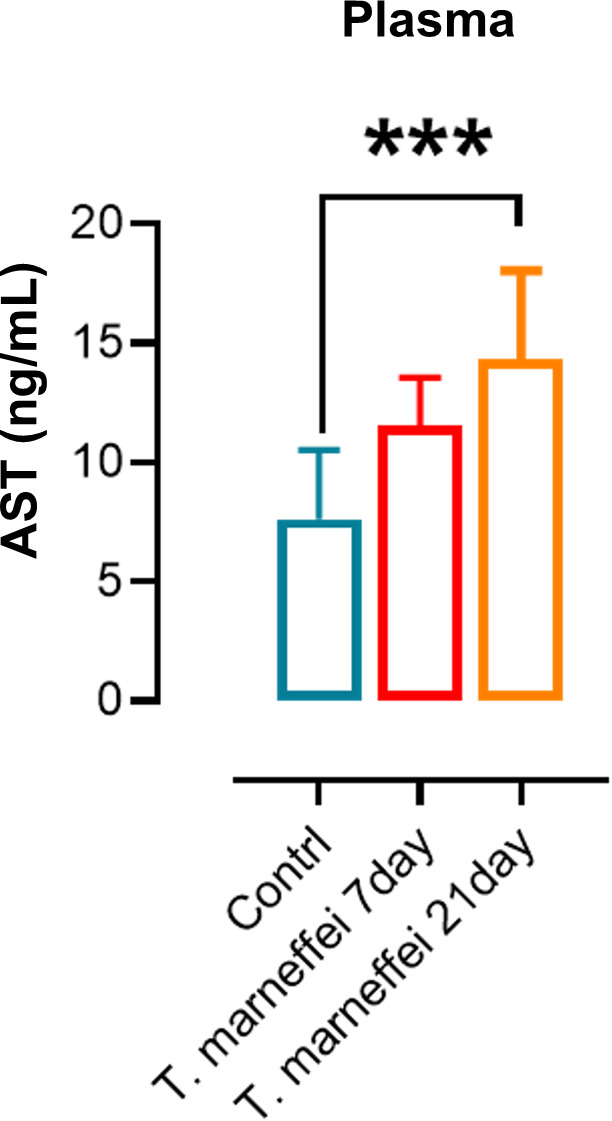
AST expression levels in the plasma of mice infected with *T. marneffei* at different time points. The x-axis represents different time points, and the y-axis represents the levels of AST. *, **P* *< 0.05; **, *P* < 0.01; ***, *P* < 0.001; ****, **P* *< 0.0001; ns, no statistical significance. Error bars: SD.

**Fig 4:**
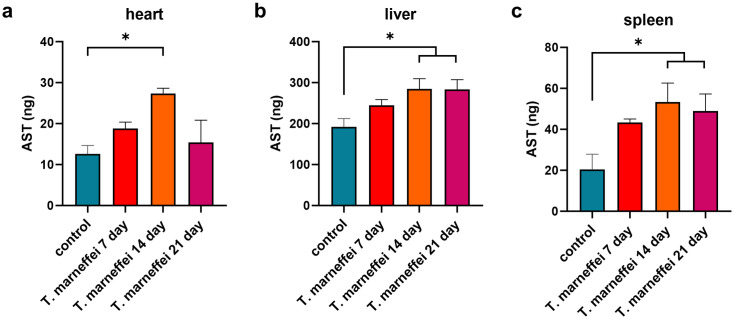
Expression levels of AST in various tissues and organs of mice infected with *T. marneffei.* The x-axis represents different tissues and organs, and the y-axis represents the levels of AST. *, **P* *< 0.05; **, *P* < 0.01; ***, *P* < 0.001; ****, **P* *< 0.0001; ns, no statistical significance. Error bars: SEM.

**Fig 5:**
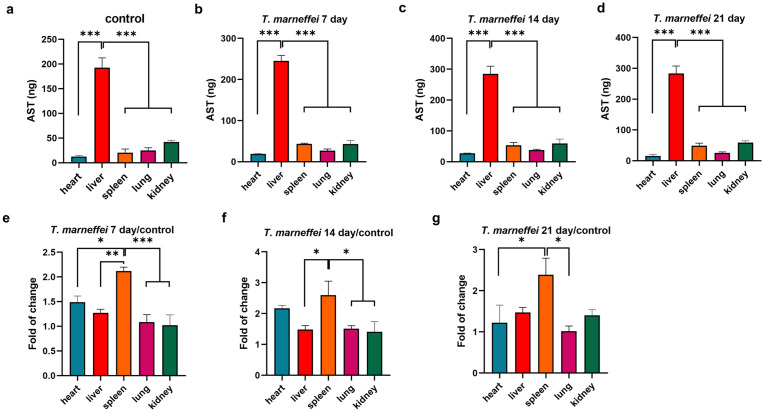
Expression levels of AST in various tissues and organs of mice infected with *T. marneffei.* The x-axis represents different tissues and organs, and the y-axis represents the levels of AST. *, **P* *< 0.05; **, *P* < 0.01; ***, *P* < 0.001; ****, **P* *< 0.0001; ns, no statistical significance. Error bars: SEM.

Plasma AST levels in *T. marneffei*- infected mice were elevated by day 7 post-infection, and remained significantly increased compared with the control group through day 21 (*P* < 0.01; Fig 3). In heart tissue, AST levels were significantly increased on day 14 but declined by day 21 (Fig 4a). AST levels in liver and spleen tissues were significantly elevated on days 14 and 21 post-infection compared to uninfected controls (Fig 4b–c). Quantitative analysis demonstrated hepatic AST levels were significantly elevated compared to extrahepatic tissues post-infection (Fig 5a–d). The spleen demonstrated the most pronounced fold-increase in AST levels among all tissues examined (Fig 5e–g).

### Hepatic and splenic activation of apoptosis, pyroptosis, and necroptosis in mice infected with *T. marneffei*

As AST release primarily indicates cellular injury and death, the observed marked AST elevation in both liver and spleen following *T. marneffei* infection suggested potential infection-induced programmed cell death. PANoptosis, which integrates features of apoptosis, pyroptosis, and necroptosis, was therefore investigated as a potential mechanism underlying the observed hepatic and splenic injury.

**Fig 6:**
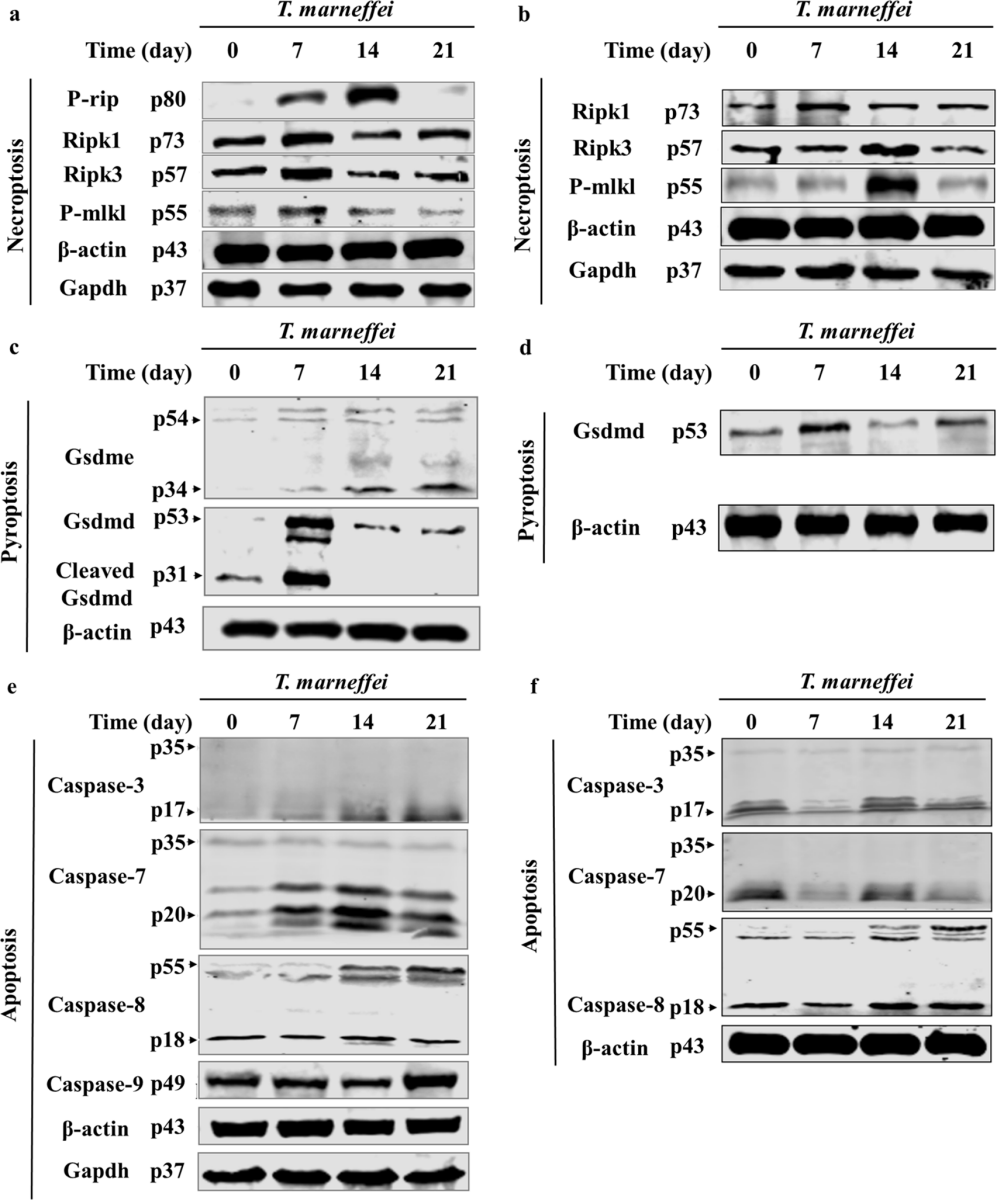
The expression levels of PANoptosis factor proteins in the liver and spleen of mice infected with *T. marneffei.* **(a-b)** Protein expression levels of necroptosis factors in liver(a) and spleen(b) in *T. marneffei* infected mice. **(c-d)** Protein expression levels of Cellular pyroptotic factors in liver(c) and spleen(d) in *T. marneffei* infected mice. **(e-f)** Protein expression levels of apoptosis factor in liver(e) and spleen(f) in *T. marneffei* infected mice.

The expression levels of necroptosis-associated proteins in liver and spleen tissues from *T. marneffei*-infected mice were analyzed. In the liver, phosphorylated RIP (P-RIP) began to increase on day 7 post-infection, peaked on day 14, and declined by day 21. RIPK1, RIPK3, and phosphorylated MLKL (P-MLKL) were elevated on day 7, but returned to baseline levels by days 14 and 21 (Fig 6a). In the spleen, RIPK1 expression increased on day 7 and normalized by day 14, while RIPK3 and P-MLKL reached peak levels on day 14 and were near baseline on day 21 (Fig 6b). Pyroptosis-associated factors were also evaluated. As shown in Fig 6c, GSDME levels in the liver progressively increased throughout the 21-day infection period. GSDMD and cleaved GSDMD levels were significantly elevated on day 7. In the spleen, GSDMD levels increased on day 7, decreased by day 14, and were nearly normal by day 21 (Fig 6d). We next assessed the expression of apoptosis-related proteins. In the liver, caspase-3 and caspase-8 levels began to rise on day 14 and peaked on day 21, showing a continuous upward trend (Fig 6e). Cleaved caspase-7 increased on day 7, peaked on day 14, and declined slightly by day 21. Caspase-9 levels were notably elevated on day 21. In the spleen, caspase-3 and caspase-7 decreased on day 7, increased on day 14, and declined again by day 21, whereas caspase-8 increased on days 14 and 21 (Fig 6f).

Together, these findings suggest that *T. marneffei* infection induces dynamic and organ-specific activation of multiple cell death pathways - including necroptosis, pyroptosis, and apoptosis - which may collectively contribute to hepatic and splenic injury during infection.

### Upregulation of inflammatory cytokines in the liver of *T. marneffei*-infected mice

**Fig 7:**
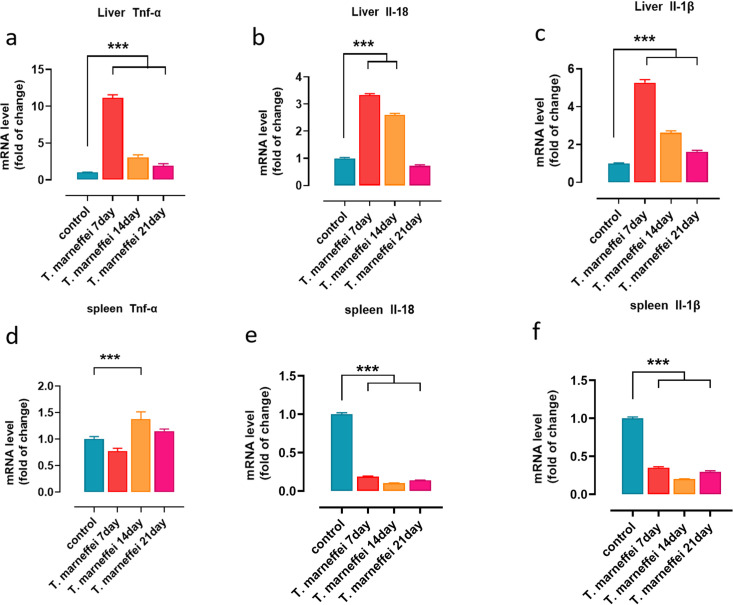
Inflammatory cytokine expression levels in the liver(a-c) and spleen(d-f) of *T.marneffei*-infected mice. The y-axis represents the fold increase in inflammatory cytokine mRNA levels, and the x-axis represents different time points. *, **P* *< 0.05; **, *P* < 0.01; ***, *P* < 0.001; ****, **P* *< 0.0001; ns, no statistical significance. Error bars: SD.

Given the detection of PANpoptosis-related factors in hepatic and splenic tissues following *T. marneffei* infection, we subsequently evaluated the expression profiles of inflammatory cytokines. Quantitative PCR was performed to assess the expression levels of Tnf-α, Il-1β, and Il-18 in liver and spleen tissues at 7, 14, and 21 days post-infection. As shown in Fig 7a - 7c, hepatic Tnf-α and Il-1β levels were significantly elevated at all three time points (days 7, 14, and 21 post-infection), with peak expression occurring at day 7. Il-18 expression was elevated on days 7 and 14, but declined by day 21. In the spleen (Fig 7d - 7f), Tnf-α was elevated only on day 14 post-infection, while both Il-1β and Il-18 showed a decreasing trend throughout the infection period. These findings suggest that *T. marneffei* infection induces a strong and sustained inflammatory response in the liver, potentially through the activation of PANoptosis.

## Discussion

*T. marneffei* is a thermally dimorphic opportunistic pathogen endemic to Southeast Asia and southern China, which causes life-threatening disseminated infections in immunocompromised hosts, particularly in individuals with advanced HIV/AIDS [20]. Among its various clinical manifestations, elevated serum AST has frequently been noted in affected individuals. However, whether this biochemical alteration represents a nonspecific consequence of immunosuppression or a pathognomonic feature of *T. marneffei*–induced organ damage remains undetermined. This study was designed to determine whether AST elevation is a specific pathological feature of *T. marneffei* infection, and to elucidate the molecular and cellular mechanisms responsible for this phenomenon.

Clinical data were analyzed to compare AST levels between HIV-infected individuals with and without *T. marneffei* co-infection. Our results demonstrated significant elevation of AST levels in the HIV/ *T. marneffei* group, independent of CD4 count and sex after propensity score matching. Importantly, AST levels decreased following antifungal therapy, supporting the notion that this elevation was reversible and infection-related. These findings suggest that AST elevation may serve as a pathophysiologically meaningful indicator of liver injury in Talaromycosis, rather than merely reflecting generalized immunosuppression.

To validate this association and investigate its pathophysiological basis, we established a mouse model of disseminated *T. marneffei* infection. Infected mice exhibited significantly elevated plasma AST levels, with the liver demonstrating the highest absolute concentrations and the spleen showing the greatest fold change. These findings mirrored clinical observations and supported the tissue-specific injury hypothesis in *T. marneffei* infection. The dominant AST release from the liver likely reflects its central metabolic role and vulnerability to inflammatory and cytotoxic insults [21,22], whereas the spleen’s response may be driven by immune cell infiltration and necrotic processes.

Beyond the AST-specific findings, our clinical data also revealed that *T. marneffei* infection is associated with a broader spectrum of hematological and biochemical abnormalities. Compared to HIV-monoinfected individuals, patients with HIV/*T. marneffei* co-infection exhibited pronounced reductions in CD4, RBC, WBC, Lym, PLT, Hb, along with decreased TP and ALB. These hematologic alterations suggest a potential manifestation of anemia in patients with *T. marneffei*, potentially reflecting the multisystemic impact of fungal dissemination. The elevations in ALT and AST/ALT ratio, together with decreased ALB, provide definitive evidence of hepatic injury. Notably, *T. marneffei* infection exhibited a higher prevalence among males and individuals in farming or labor-intensive occupations, echoing the known environmental reservoirs of the fungus and pointing to social and occupational exposure risks [23–25]. These findings not only corroborate the AST-related observations but also highlight that *T. marneffei* is capable of both organ-specific and systemic.

Mounting evidence suggests that AST elevation in infectious contexts frequently reflects immune-mediated hepatocyte damage. Acetaminophen and D-galactosamine hepatotoxicity models exhibit comparable transaminase elevations associated with cell death pathways [26,27]. These parallels prompted us to explore whether *T. marneffei*–induced tissue injury involves inflammatory programmed cell death.

PANoptosis is a complex inflammatory programmed cell death pathway involving multiple regulatory mechanisms that integrate pyroptosis, apoptosis, and necroptosis [28,29]. Pyroptosis is mainly mediated by inflammasome-induced activation of caspase-1 [30]. Apoptosis occurs primarily via death receptor signaling and mitochondrial outer membrane permeabilization, leading to controlled cellular dismantling [31]. Necroptosis is executed through the RIPK1-RIPK3-MLKL signaling axis, which disrupts plasma membrane integrity [32]. Recent studies have established that PANoptosis plays a critical and sophisticated role in host cell death during microbial infections [33–35]. Experimental evidence shows that Z-DNA binding protein 1 (ZBP1) induces PANoptosis in the dimorphic fungus *Candida albicans* [36]. Whether *T. marneffei* similarly utilizes PANoptosis to mediate hepatocyte death remains to be systematically investigated. Our findings provide initial insight into this possibility, and further mechanistic studies are warranted.

In the present study, liver tissues from *T. marneffei*-infected mice demonstrated upregulated expression of apoptosis-related factors (caspase-3, caspase-7, caspase-8, and caspase-9), pyroptosis-related factors (GSDMD, cleaved GSDMD, and GSDME), and necroptosis-related factors (P-RIP, RIPK1, RIPK3, and P-MLKL). These results suggest that *T. marneffei* infection activates PANoptotic pathways simultaneously, contributing to hepatocyte death and subsequent AST release.

Interestingly, splenic tissue exhibited a different pattern. While necroptosis-associated markers demonstrated significant elevatiom, pyroptosis and apoptosis markers remained unchanged. This tissue-specific difference in PANoptotic activation could be due to differential fungal tropism, immune cell populations, or organ-specific inflammatory environments. In particular, the liver’s unique microenvironment, including resident macrophages and high metabolic activity, may predispose it to PANoptosis-mediated injury under systemic fungal challenge.

Inflammatory signaling was further assessed by evaluating the expression of cytokines levels in liver and spleen tissues. We found that Tnf-α, Il-1β, and Il-18 were markedly upregulated in liver tissue, especially in the early phase of infection, supporting a role for robust local inflammation in mediating hepatocellular injury. These cytokines are known to amplify and be amplified by PANoptotic processes [34,37]. In contrast, the spleen displayed suppressed expression of Il-1β and Il-18 throughout the course of infection, despite demonstrating biochemical markers of injury. This discordance may be indicative of immune evasion strategies employed by *T. marneffei* in secondary lymphoid organs, or the activation of compensatory anti-inflammatory responses to avoid immunopathology [38–40].

Collectively, our findings support a model in which *T. marneffei* infection induces liver-specific PANoptotic, accompanied by cytokine amplification and marked AST release. The differential involvement of cell death pathways and inflammatory responses in the liver versus the spleen underscores the complexity of host-pathogen interactions during systemic fungal infections.

In addition, Previous studies have applied the Damage Response Framework (DRF) to elucidate the pathogenicity of *T. marneffei* infection [41]. The DRF conceptualizes infection outcomes as a spectrum of host-microbe interactions, where varying intensities of host responses can lead to either protective effects or pathological damage. In our study, we observed that *T. marneffei* infection induces PANoptosis, which closely correlates with hepatocyte death. This may suggest a stage-dependent dual role for PANoptosis in the pathogenesis of *T. marneffei*: during early infection, PANoptosis may facilitate protective clearance of infected hepatocytes, while persistent PANoptosis at later stages could exacerbate immunopathology and contribute to liver injury. The DRF provides a conceptual basis for understanding host-pathogen outcome variability. Nevertheless, emerging evidence suggests that *T. marneffei* EVs can robustly activate inflammatory responses in immune cells [42,43]. However, their direct role in regulating hepatocyte death, including PANoptosis, remains to be elucidated. The EVs may act as critical mediators of PANoptosis during *T. marneffei* infection. Future studies employing multi-omics approaches to characterize EVs cargo composition, combined with functional assays, could systematically assess their regulatory effects on key PANoptosis components such as ZBP1, RIPK3, and caspase cascades.

This study has several limitations. The key regulatory factors and detailed molecular mechanisms underlying PANoptosis following *T. marneffei* infection remain incompletely understood. Future work will focus on systematically elucidating the molecular regulatory network by integrating multi-omics analyses with functional cellular experiments. Additionally, this study did not extensively examine changes in damage-related markers (e.g., AST, ALT) and PANoptosis effector molecules across multiple tissues such as liver and spleen in infected patients. To strengthen and validate our findings, we plan to collaborate with clinical institutions to establish a well-characterized patient cohort for comprehensive future investigations. Moreover, the precise timing of the conidia-to-fission yeast transition in the murine infection model was not explicitly determined. Future studies will employ histopathological staining techniques and yeast-phase-specific markers or antibodies to better define this critical transformation timeline.

## Conclusion

In summary, this study bridges clinical and experimental evidence to demonstrate that *T. marneffei* infection induces liver injury, characterized by elevated AST levels tthat are reversible after antifungal treatment. Thus, AST may serve as a useful auxiliary biomarker for evaluating hepatic involvement and monitoring therapeutic response in Talaromycosis. Mechanistically, the elevation of AST is closely associated with PANoptosis-mediated hepatocellular death, revealing a key pathological process underlying organ damage in *T. marneffei* infection. These findings enhance our understanding of fungal pathogenesis and provide a theoretical basis for developing diagnostic and therapeutic strategies targeting inflammatory cell death pathways in the context of HIV-associated opportunistic infections.

## Supporting information

S1 Table Sequences of primers. (DOCX)

S2 Table Basic characteristics of the study population. (DOCX)
