# Supplementary material for: PANoptosis-mediated mechanisms underlying AST elevation in Talaromyces marneffei infection
Source: PLoS Negl Trop Dis. 2025 Sep 2;19(9):e0013443. doi: 10.1371/journal.pntd.0013443 (PMC12404362; doi:10.1371/journal.pntd.0013443)
Supplement: S1 Table — (DOCX) [file pntd.0013443.s001.docx]

| S1 Table. Sequences of primers | | |
| --- | --- | --- |
| Gene Name | Primer Direction | Primer Sequence (5'→3') |
| Gapdh | Forward | GCTCCAAAGGACTTGTACGTG |
|  | Reverse | TGATCTGAAGGGCAGCATTTC |
| Caspase-3 | Forward | CCACAGTGCAGCTACCTCAA |
|  | Reverse | AAGCTCTGTTTGACAACTGTATATT |
| Caspase-6 | Forward | CGGATGCAATCGGCAAGAAG |
|  | Reverse | GACCAAGTCAAATAGGCCCAC |
| Caspase-7 | Forward | TGGGACTTTTGCTTTCAGTTTTCCC |
|  | Reverse | TCTTCTCGTTGAAGCGTGGAT |
| Caspase-8 | Forward | AAGCAGGAAGTGTGAGAGGC |
|  | Reverse | CATCCTCGATGGTCTCCTGC |
| Caspase-9 | Forward | ACCTTCCCAGGTTGCCAATG |
|  | Reverse | CTGCTCCACATGCCCTACA |
| Tnf-α | Forward | CAGGCGGTGCCTATGTCTC |
|  | Reverse | CGATCACCCCGAAGTTCAGTAG |
| IL-6 | Forward | CTGCAAGAGACTTCCATCCAG |
|  | Reverse | AGTGGTATAGACAGGTCTGTTGG |
| IL-10 | Forward | GCTGGACAACATACTGCTAACC |
|  | Reverse | ATTTCCGATAAGGCTTGGCAA |
| IL-18 | Forward | GACTCTTGCGTCAACTTCAAGG |
|  | Reverse | CAGGCTGTCTTTTGTCAACGA |
| Ripk1 | Forward | AACTGACTAAGTGGCACCCG |
|  | Reverse | CGCTCCATGAGGCTGATGAT |
| Ticam1 | Forward | CCCATTGAATGCACAGAGTTGTC |
|  | Reverse | GAGTGGTCCAAAACAGGGGA |
| Ripk3 | Forward | GCCTTCCTCTCAGTCCACAC |
|  | Reverse | CTCACCAGAGGAACCGCATA |
