# Supplementary material for: PANoptosis-mediated mechanisms underlying AST elevation in Talaromyces marneffei infection
Source: PLoS Negl Trop Dis. 2025 Sep 2;19(9):e0013443. doi: 10.1371/journal.pntd.0013443 (PMC12404362; doi:10.1371/journal.pntd.0013443)
Supplement: S2 Table — (DOCX) [file pntd.0013443.s002.docx]

| S2 Table. Basic characteristics of the study population | | | |
| --- | --- | --- | --- |
| Variables | Features | Number (n) | Proportion (%) |
| Sex | Male | 87 | 69.60 |
|  | Female | 38 | 30.40 |
| Nationality | Han | 57 | 45.60 |
|  | Zhuang | 61 | 48.80 |
|  | Other | 7 | 5.60 |
| Occupation | Farmer and Worker | 79 | 63.20 |
|  | Self-employed individual | 11 | 8.80 |
|  | Unemployed | 23 | 9.20 |
|  | Managers, Office clerks, and Students | 10 | 8.00 |
|  | Other | 2 | 1.60 |
| CD4 lymphocyte count | <100/μL | 90 | 72.00 |
|  | <200/μL | 102 | 81.60 |
